# Rapamycin/metformin co‐treatment normalizes insulin sensitivity and reduces complications of metabolic syndrome in type 2 diabetic mice

**DOI:** 10.1111/acel.13666

**Published:** 2022-08-19

**Authors:** Peter C. Reifsnyder, Kevin Flurkey, Rosalinda Doty, Nigel A. Calcutt, Robert A. Koza, David E. Harrison

**Affiliations:** ^1^ The Jackson Laboratory Bar Harbor Maine USA; ^2^ Department of Pathology University of California San Diego La Jolla California USA; ^3^ Center for Molecular Medicine Maine Medical Center Research Institute Scarborough Maine USA; ^4^ Graduate School of Biomedical Sciences and Engineering University of Maine Orono Maine USA; ^5^ Pennington Biomedical Research Center Baton Rouge Louisiana USA

**Keywords:** diabetic complications, insulin sensitivity, metformin, mice, rapamycin, type 2 diabetes

## Abstract

Rapamycin treatment has positive and negative effects on progression of type 2 diabetes (T2D) in a recombinant inbred polygenic mouse model, male NONcNZO10/LtJ (NcZ10). Here, we show that combination treatment with metformin ameliorates negative effects of rapamycin while maintaining its benefits. From 12 to 30 weeks of age, NcZ10 males were fed a control diet or diets supplemented with rapamycin, metformin, or a combination of both. Rapamycin alone reduced weight gain, adiposity, HOMA‐IR, and inflammation, and prevented hyperinsulinemia and pre‐steatotic hepatic lipidosis, but exacerbated hyperglycemia, hypertriglyceridemia, and pancreatic islet degranulation. Metformin alone reduced hyperinsulinemia and circulating c‐reactive protein, but exacerbated nephropathy. Combination treatment retained the benefits of both while preventing many of the deleterious effects. Importantly, the combination treatment reversed effects of rapamycin on markers of hepatic insulin resistance and normalized systemic insulin sensitivity in this inherently insulin‐resistant model. In adipose tissue, rapamycin attenuated the expression of genes associated with adipose tissue expansion (*Mest, Gpam*), inflammation (*Itgam*, *Itgax*, *Hmox1, Lbp*), and cell senescence (*Serpine1*). In liver, the addition of metformin counteracted rapamycin‐induced alterations of *G6pc, Ppara*, and *Ldlr* expressions that promote hyperglycemia and hypertriglyceridemia. Both rapamycin and metformin treatment reduced hepatic *Fasn* expression, potentially preventing lipidosis. These results delineate a state of “insulin signaling restriction” that withdraws endocrine support for further adipogenesis, progression of the metabolic syndrome, and the development of its comorbidities. Our results are relevant for the treatment of T2D, the optimization of current rapamycin‐based treatments for posttransplant rejection and various cancers, and for the development of treatments for healthy aging.

## Graphical abstract

Rapamycin and metformin combination treatment in a mouse model of type 2 diabetes, NONcNZO10/LtJ males, prevents hyperinsulinemia, normalizes insulin sensitivity, and reduces pathological complications of diabetes. When combined, each compound prevents the negative side effects of the other. These results are relevant for the treatment of diabetes, the optimization of current rapamycin‐based treatments, and the development of treatments for healthy aging.

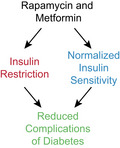

**Abbreviations**
ACR: albumin/creatinine ratio
ANOVA: analysis of variance
APOB: Apolipoprotein B
APOE: Apolipoprotein E
BKS‐*db/db*
: C57BLKS/J‐*Lepr* ^*db*^
BMD: bone mineral density
BW: body weight
Chol.: cholesterol
CRP: c‐reactive protein
Dio‐2: type II iodothyronine deiodinase
DXA: dual X‐ray Absorptiometry
EPI: epididymal
Fasn: fatty acid synthase
G6pc: glucose‐6‐phosphatase
Gck: glucokinase
Glut4: glucose transporter member 4
Gpam: glycerol‐3‐phosphate acyltransferase, mitochondrial
HbA1c: hemoglobin A1C
HDLD: high‐density lipoprotein
Hmox1: heme oxygenase 1
HOMA‐IR: Homeostatic Model Assessment for insulin resistance
IENF: intra‐epidermal nerve fiber
Igf‐1: insulin‐like growth factor 1
ING: inguinal
Itgam: integrin alpha M
Itgax: integrin alpha X
ITT: insulin tolerance test
Lbp: lipopolysaccharide binding protein
Lep: leptin
Ldlr: low‐Density lipoprotein
MANOVA: multivariate analysis of variance
Mest: mesoderm‐specific transcript
MET: metformin
mTORC: mechanistic Target of Rapamycin
NcZ10: NONcNZO10/LtJ
NEFA: non‐esterified fatty acids
  PG
  plasma glucose
PIC: pancreatic insulin content
Ppara: peroxisome proliferator‐activated receptor alpha
Ppargc1a: peroxisome proliferator‐activated receptor gamma coactivator 1‐alpha
RAPA: rapamycin
RAPA/MET: combination of rapamycin and metformin
Serpine1: plasminogen activator inhibitor 1 (aka PAI‐1)
Slc2a4: solute carrier family 2 (aka Glut4)
T2D: type 2 diabetes
TG: triglycerides
Ucp1: uncoupling protein 1
UNT: untreated

## 1 INTRODUCTION

Rapamycin increases lifespan reliably in numerous organisms (Harrison et al., 2009; Arriola Apelo & Lamming, 2016; reviewed in Gomez‐Linton et al., 2019) via inhibition of mTORC (mechanistic Target Of Rapamycin Complex), a molecular complex that regulates growth and metabolism. In mice, rapamycin increases lifespan and delays the onset of multiple age‐related diseases (Wilkinson et al., 2012), suggesting that it retards basic mechanisms of aging. In humans, rapamycin is widely used as an immune suppressant after organ transplantation, and as an anticancer agent. However, it promotes hyperglycemia in roughly 15% of human patients (Barlow et al., 2013), presumably by promoting systemic and hepatic insulin resistance and diminishing pancreatic response to glucose (Houde et al., 2010; Lamming et al., 2012; Schindler et al., 2014; Yang et al., 2012). In healthy adult mice, rapamycin treatment typically delays glucose clearance and can promote insulin resistance, depending on strain and experimental protocol (Lamming et al., 2013; Yang et al., 2012). Yet, in a number of mouse inbred strains, including mouse models of type 2 diabetes (T2D), when rapamycin is administered through the diet, it ameliorates multiple T2D‐driven impairments and either has no effect on insulin resistance, or actually promotes insulin sensitivity, even while exacerbating hyperglycemia (Lamming et al., 2013; Reifsnyder et al., 2014, 2016, 2018, 2020; Yang et al., 2012). We have investigated the rapamycin‐driven mitigation of T2D comorbidities in the presence of hyperglycemia using male NONcNZO10/LtJ (NcZ10) mice, a model of human age‐related T2D featuring polygenic adult‐onset hyperglycemia driven by moderate obesity with insulin resistance (Leiter et al., 2013; Leiter & Reifsnyder, 2004; Reifsnyder & Leiter, 2002). In this model, long‐term (>3 month) treatment with rapamycin in the diet improves systemic insulin sensitivity and reduces diabetic nephropathy despite elevating diabetic hyperglycemia (Reifsnyder et al., 2014).

To explore the potential to modulate the glycemic influence of rapamycin, in this study, we evaluated combined rapamycin‐metformin treatment in NcZ10 males. Metformin is a first‐line antihyperglycemic medication commonly prescribed for type 2 diabetes. Previous studies have shown that this combination treatment in a genetically heterogeneous stock of mice (HET3) increased median lifespan by 23% and maximum lifespan by ~14% for both sexes (Strong et al., 2016). In young normal Sprague–Dawley rats, short‐term treatment (4 weeks, s.c. injection) with the combination prevented the impaired glucose tolerance and hyperglycemia elicited by rapamycin treatment alone (Jin et al., 2017; Sun et al., 2019). In high fat‐fed DBA/1J mice, metformin (10 weeks, per os) also prevented rapamycin‐induced (per os) hyperglycemia and the associated impaired glucose tolerance (Kim et al., 2020). In long‐term studies with HET3 mice, the combination treatment, given in the diet, improved rapamycin‐induced impaired glucose tolerance after 3 months of treatment for females, but for males, it had no effect on rapamycin‐induced impaired glucose tolerance after 9 months of treatment (Weiss et al., 2018). Thus, although metformin modulates the effects of rapamycin treatment on glucose metabolism, specific results vary across studies, demonstrating that experimental design and the model used influence this interaction.

We assessed the effects of long‐term dietary treatment with rapamycin, metformin, or their combination using the NcZ10 mouse, a strain developed to model human populations most vulnerable to T2D. Feeding mimics the oral treatment mode used for humans. Our objective was to identify potential physiological mechanisms that determine beneficial and detrimental outcomes of these treatments.

## 2 RESULTS

We examined progression of diabetogenic hyperglycemia and associated pathogenic phenotypes in male NcZ10 mice maintained on either 11% fat chow diet (untreated) or the same diet supplemented with 14 ppm rapamycin (RAPA), 0.1% metformin (MET) or a combination of both drugs (RAPA/MET) from 12 to 30 weeks of age. We evaluated results for 10 individual phenotypic categories that govern the development and pathogenesis of T2D: *Obesity*, a key initiating factor in the development of T2D, is associated with elevated *circulating lipids* and the initiation of *adipose‐derived inflammatory processes*, which are important drivers of *systemic insulin resistance*. This leads to chronic *hyperinsulinemia* and *hyperglycemia*, which promote the pathogenic consequences of T2D, including *hepatic lipidosis*, *pancreatic impairment, nephropathy*, and *neuropathy*.

### 2.1 Obesity

**FIGURE 1:**
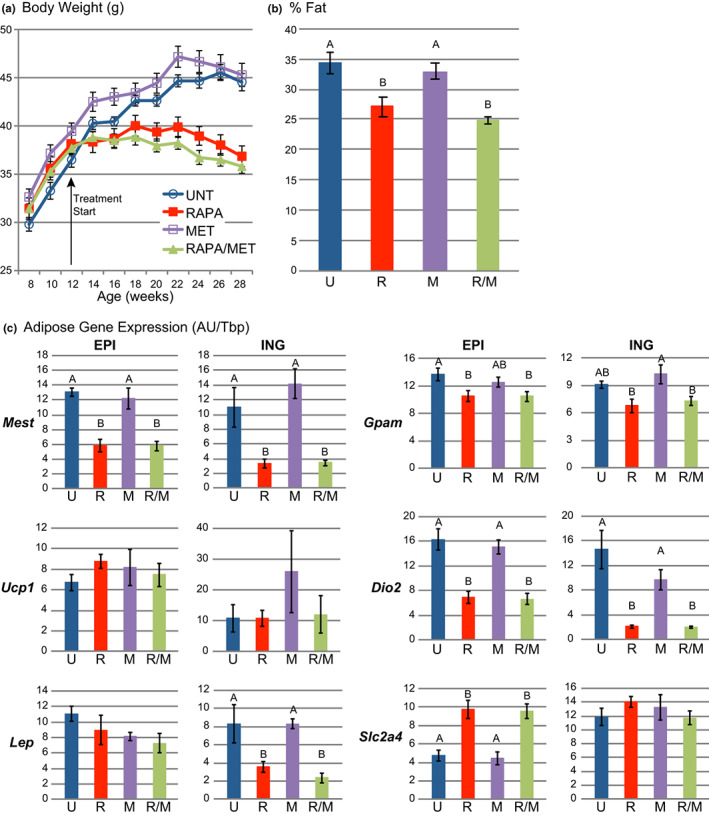
RAPA‐ and RAPA/MET‐treatments reduced obesity. (a) RAPA‐ and RAPA/MET‐treatments prevented adult weight gain typical of NcZ10 males (*p* < 0.0001, repeated measures MANOVA for both RAPA and RAPA/MET vs. UNT). MET‐ treatment had no effect. (b) RAPA‐ and RAPA/MET‐treatments significantly reduced body fat, determined by DXA. (c) RAPA‐ and RAPA/MET‐treatments reduced *Mest* and *Gpam* expression in both fat pads. While *Ucp1* expression was unaffected, RAPA‐ and RAPA/MET‐treatments reduced *Dio2* in both fat pads. Treatment effects on *Lep* expression mirrored effects on fat pad weight in both fat pads. RAPA‐ and RAPA/MET‐treatments increased *Slc2a4* expression in EPI, but not ING fat. Values are mean ± SE. All multiple comparisons are significant at *p* ≤ 0.05 (Tukey‐ Kramer). *N* = 9–10 mice per treatment except *N* = 4 mice per treatment for % Fat and ING fat gene expression. Within each histogram, treatment groups not annotated by the same superscript letter are significantly different at *p* < 0.05 (Tukey–Kramer HSD). Untreated = UNT and U, RAPA‐treated = RAPA and R, MET‐treated = MET and M, RAPA/MET‐treated = RAPA/MET and R/M

RAPA‐ and RAPA/MET‐treatment halted the normal body weight gain seen in untreated mice (Figure 1a, Table 1), primarily by preventing fat mass expansion (Figure 1b, Table 1). By the end of the study, after 18 weeks of treatment, RAPA‐ and RAPA/MET‐treated mice showed reduced adiposity (% Fat by DXA) typified by reduced subcutaneous inguinal (ING) adipose tissue (Figure 1b, Table 1). However, this reduction was depot‐specific; visceral epididymal (EPI) adipose tissue was unaffected by any treatment. MET‐treatment alone did not affect body weight or composition. Longitudinal growth (anus to nose length), bone mineral density, and food intake (Table 1) did not differ among treatment groups.

**TABLE 1:** Effects of RAPA‐, MET‐, and RAPA/MET‐treatments in type 2 diabetic NcZ10 mice: Body composition and food intake

Group	UNT	RAPA	MET	RAPA/MET
BW at sacrifice (g)	42.9 ± 0.8	36.0 ± 1.0***	44.5 ± 1.2	34.6 ± 0.7***
ΔBW 12–28 wks (g)	+8.3 ± 1.0	−1.3 ± 1.1***	+5.9 ± 0.7	−2.3 ± 0.6***
Food intake (kcal/d/kg lean wt)	343 ± 26	418 ± 21	393 ± 25	385 ± 27
EPI fat Wt. (g)	1.18 ± 0.10	1.18 ± 0.09	1.25 ± 0.10	1.06 ± 0.07
EPI fat Wt./BW (%)	2.7 ± 0.2	3.3 ± 0.2	2.8 ± 0.3	3.1 ± 0.1
ING fat Wt. (g)	0.61 ± 0.09	0.27 ± 0.03**	0.64 ± 0.06	0.24 ± 0.03**
ING fat Wt./BW (%)	1.5 ± 0.2	0.8 ± 0.1**	1.4 ± 0.1	0.7 ± 0.1**
Fat mass (g) by DXA	15.5 ± 1.2	10.1 ± 0.9**	14.8 ± 0.7	8.9 ± 0.5**
% Fat by DXA	34.4 ± 1.8	27.1 ± 1.6*	33.0 ± 1.3	24.9 ± 0.6**
Lean mass (g) by DXA	29.5 ± 1.0	26.9 ± 0.5	30.0 ± 0.8	26.8 ± 0.6
% Lean by DXA	65.6 ± 1.8	72.9 ± 1.6*	67.0 ± 1.3	75.0 ± 0.6**
Anus/nose length (cm)	9.7 ± 0.1	9.8 ± 0.1	9.9 ± 0.1	9.6 ± 0.1
BMD (g/cm^2^) (DXA)	0.055 ± 0.001	0.056 ± 0.001	0.057 ± 0.001	0.055 ± 0.001

*Note*: Mean ± SE. ANOVA followed by Tukey–Kramer HSD, significant effects indicated for pairwise comparisons of untreated to treated groups: ***p ≤0.0001, **p ≤ 0.01, *p ≤ 0.05. *N* = 9–10 for BW, ΔBW, EPI Wt., EPI Wt./BW, and Anus/nose length; *N* = 3–4 for ING Wt., ING Wt./BW, Food Intake, and DXA. Untreated = UNT, RAPA‐treated = RAPA, MET‐treated = MET, RAPA/MET‐treated = RAPA/MET.

Gene expression analyses of both EPI and ING adipose tissue (Figure 1c, Table S1) indicated robust RAPA‐ and RAPA/MET‐mediated downregulation of *Mest* (mesoderm‐specific transcript) and *Gpam* (glycerol‐3‐phosphate acyltransferase, mitochondrial), genes associated with adipose tissue function, growth, and expansion. Thermogenesis‐associated genes *Ucp1, Ppargc1α*, and *Dio2* were unaffected or downregulated by RAPA‐treatment (Table S1).

*Lep* (leptin) expression reflected treatment effects on adipose tissue weight: *Lep* expression was reduced 60–70% in the ING fat of RAPA‐ and RAPA/MET‐treated mice but unaffected in their EPI fat (Table 1). *Slc2a4* (solute carrier family 2, facilitated glucose transporter member 4; Glut4) was upregulated in EPI fat but not in ING fat. MET‐treatment alone had minimal effects on gene expression in adipose tissue (Figure 1c, Table S1).

### 2.2 Circulating lipids

**FIGURE 2:**
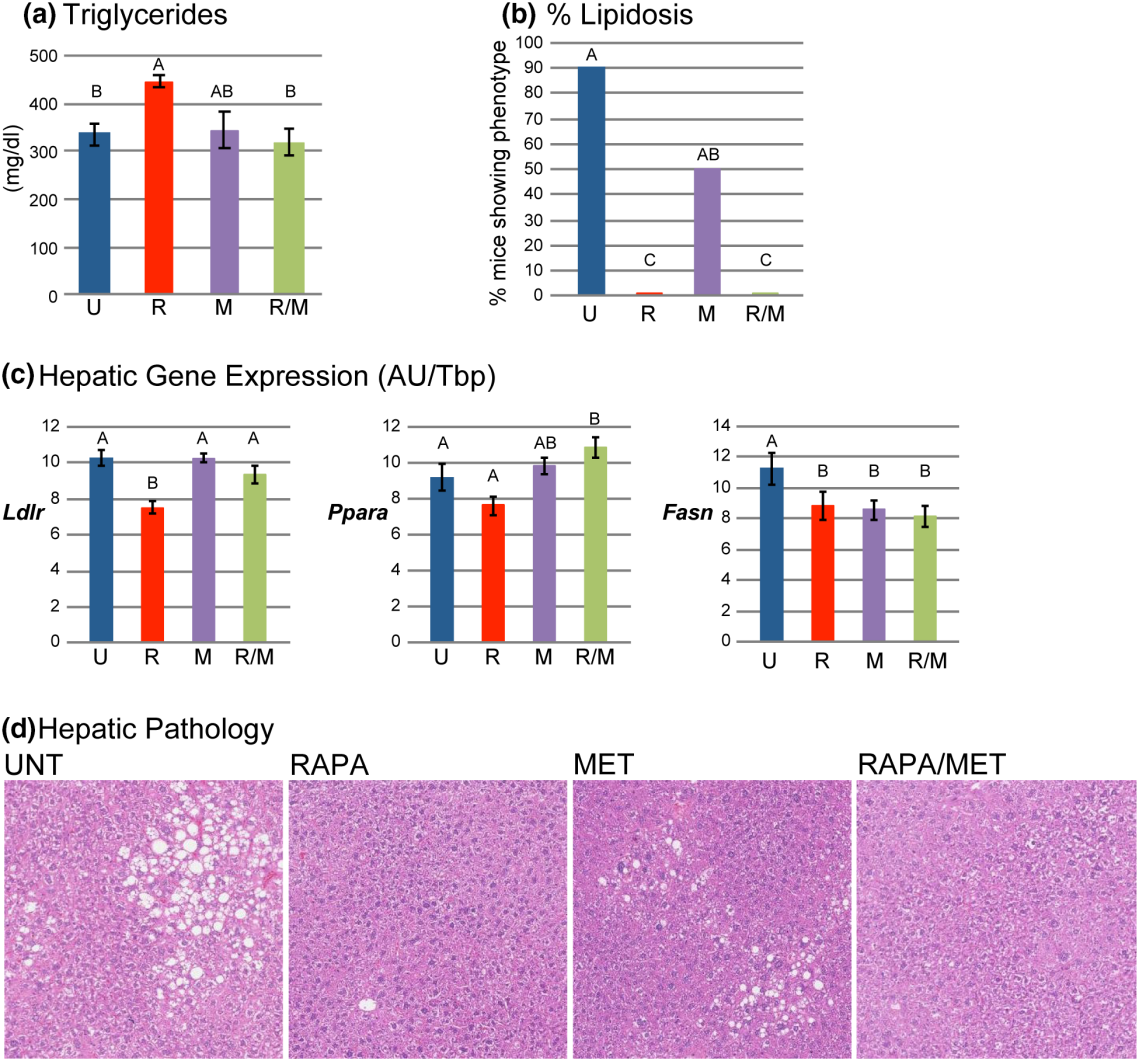
RAPA/MET‐treatment prevented RAPA‐mediated elevation of triglycerides. RAPA‐ and RAPA/MET‐treatments prevented hepatic lipidosis. (a) RAPA‐treatment increased plasma triglyceride level, but combination with MET blocked this effect. (b) Reduction of hepatic lipidosis was suggested in MET‐treated mice; hepatic lipidosis was completely prevented in RAPA‐ and RAPA/MET‐treated mice (nominal logistic fit, likelihood ratio test for presence of lipidosis: overall, *p* < 0.0001; post hoc pairwise tests: UNT vs MET, *p* = 0.06; UNT vs RAPA or RAPA/MET, *p* < 0.0001). (c) RAPA‐treatment reduced hepatic *Ldlr* and *Ppara* expression; co‐treatment with MET blocked the reduction of *Ldlr* expression and increased *Ppara* expression consistent with triglyceride levels. All 3 treatments reduced expression of *Fasn*. (d) Representative histology of moderate hepatic lipidosis in UNT mice, mild hepatic lipidosis in MET‐treated mice, and absence of hepatic lipidosis in RAPA‐ and RAPA/MET‐treated mice. Mean ± SE. *N* = 6–8 mice per treatment for triglycerides and 9–10 per treatment for gene expression. Significance annotations and treatment abbreviations as in Figure 1

RAPA‐treatment for 18 weeks elevated the level of circulating triglycerides above the preexisting hypertriglyceridemia while MET‐treatment alone did not affect circulating triglycerides. Importantly, combination RAPA/MET‐treatment prevented the RAPA‐mediated elevation of circulating triglycerides (Figure 2a, Table 2). Total circulating cholesterol, HDLD cholesterol, and nonesterified fatty acids were unaffected by RAPA‐ or MET‐treatment, whereas combination RAPA/MET‐treatment significantly lowered HDLD cholesterol (Table 2), which typically constitutes 70%–80% of total circulating cholesterol in mice.

**TABLE 2:** Effects of RAPA‐, MET‐, and RAPA/MET‐treatment in type 2 diabetic NcZ10 mice: Health and functional test results at end of study

Group	UNT	RAPA	MET	RAPA/MET
TG (mg/dl)	336 ± 22	445 ± 13**	344 ± 38	319 ± 28
Total Chol. (mg/dl)	144 ± 7	160 ± 5	132 ± 6	135 ± 10
HDLD Chol. (mg/dl)	114 ± 5	126 ± 3	106 ± 5	97 ± 4*
NEFA (mEq/L)	1.8 ± 0.2	2.3 ± 0.1	1.5 ± 0.1	2.0 ± 0.1
Insulin (ng/ml)	3.7 ± 0.5	1.4 ± 0.1***	2.5 ± 0.4*	1.1 ± 0.1***
HOMA‐IR index	72.6 ± 35.7	38.2 ± 4.5**	51.3 ± 9.0	23.8 ± 4.0***
IGF‐1 (ng/ml)	589 ± 32	532 ± 25	517 ± 20	453 ± 22**
Hepatic lipidosis^‡^	9/10	0/9***	5/10*	0/9***
IENF (profiles/mm)	12.2 ± 1.9	12.3 ± 1.0	11.4 ± 1.2	11.9 ± 1.4

*Note*: Mean ± SE. ANOVA followed by Tukey–Kramer HSD, significant effects indicated for pairwise comparisons of untreated to treated groups: ***p ≤0.0001, **p ≤ 0.01, *p ≤ 0.05. *N* = 6–10. ^‡^Presence/absence of lipidosis evaluated by nominal logistic regression, overall effect, *p* < 0.0001. Untreated = UNT, RAPA‐treated = RAPA, MET‐treated = MET, RAPA/MET‐treated = RAPA/MET, TG = triglycerides; Chol. = cholesterol; NEFA = non‐esterified fatty acids; IENF = intra‐epidermal small sensory nerve fiber density. Insulin and HOMA‐IR were assessed at 12 weeks of treatment, IGF‐1 at 16 weeks of treatment, all others at 17–18 weeks of treatment.

The elevation of circulating triglycerides in RAPA‐treated mice corresponds with their reduced hepatic expression of *Ldlr* mRNA. The restoration, in RAPA/MET‐treated mice, of *Ldlr* expression was consonant with their elevation of *Ppara* expression, which positively regulates *Ldlr*, and with the prevention of RAPA‐induced hypertriglyceridemia (Figure 2c, Table S2).

### 2.3 Hepatic lipidosis

We observed mild‐to‐moderate hepatic lipidosis in 9/10 untreated NcZ10 males. While MET‐treatment alone had a modest effect, hepatic lipidosis was completely prevented by RAPA‐ and RAPA/MET‐treatment (Figure 2b,d, Table 2). Insulin‐sensitive hepatic *Fasn* expression is closely associated with the development of lipidosis leading to steatosis (Dorn et al., 2010); the reduction in hepatic *Fasn* expression by all three treatments paralleled the reduction in circulating insulin and hepatic lipidosis.

### 2.4 Inflammation

**FIGURE 3:**
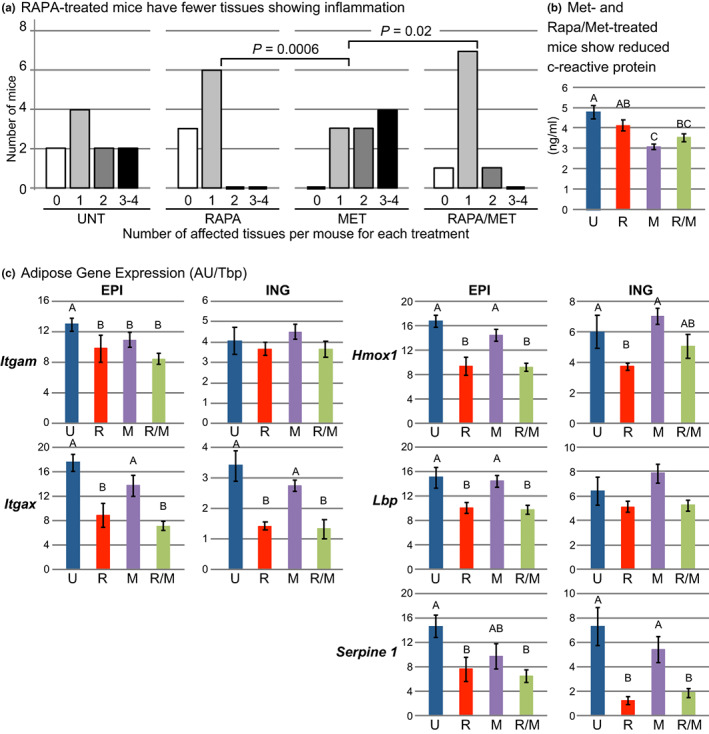
RAPA‐treatment reduced extent of inflammation. Each mouse was scored (0–4) for number of tissues (pancreas, liver, kidney, and spleen) with histologic evidence of inflammation. For analysis, numbers of mice with 3 or 4 affected tissues were combined. (a) Treatment significantly affected the extent of inflammation (*p* = 0.0006, ordinal logistic fit, likelihood ratio test). MET‐treated mice had the greatest extent of inflammation. RAPA‐ and RAPA/MET‐treatments significantly reduced the extent of inflammation compared to MET‐treatment (post hoc pairwise analysis, ordinal logistic fit). (b) c‐reactive protein is an indication of the intensity of systemic inflammation. Plasma c‐reactive protein was reduced in MET‐ and RAPA/MET‐treated mice. (c) RAPA‐ and RAPA/MET‐treatments reduced *Itgam* and *Itgax* expression in EPI fat and *Itgax* expression in ING fat, indicative of diminished myeloid cell infiltration possibly mediated in part by reduced *Hmox1* expression. *Lbp* and *Serpine1* expression was reduced by both RAPA‐ and RAPA/MET‐treatments in EPI fat, and *Serpine1* expression was also reduced by both treatments in ING fat. Mean ± SE. *N* = 9–10 mice per treatment except *N* = 4 per treatment for ING fat gene expression. Significance annotations and treatment abbreviations as in Figure 1

We evaluated systemic inflammation histologically by identifying hyperplasia of the spleen and foci of lymphocytes in the pancreas, liver, and kidney, and by assay of the circulating level of the acute phase protein, c‐reactive protein (CRP). As observed previously for NcZ10 mice (Reifsnyder & Leiter, 2002), pancreatic peri‐insulitis and peri‐vasculitis were present at 30 weeks, after 18 weeks of RAPA‐treatment (79% of both treated and untreated NcZ10 mice). Inflammation incidence among other tissues in NcZ10 mice ranged from 14%–26%. Overall, MET‐treated mice had the greatest extent of inflammation, whereas RAPA‐ and RAPA/MET‐treatment significantly diminished the extent of inflammation compared with MET‐treatment alone (Figure 3a). In contrast, circulating CRP, a marker for intensity of systemic inflammation, was not significantly affected by RAPA‐treatment, but it was significantly reduced by both MET, and RAPA/MET‐treatment. (Figure 3b). Thus, rapamycin and metformin appear to diminish different facets of inflammation (extent and intensity); effects on both facets are fully expressed by the combination treatment.

In adipose tissue, we used selective gene expression analysis to evaluate inflammation in more detail. *Itgam* (integrin alpha M) and *Itgax* (integrin alpha X) are genes for cell surface proteins on myeloid cells, including macrophages, monocytes, and granulocytes (Solovjov et al., 2005). RAPA‐ and RAPA/MET‐treatment diminished *Itgax* and *Itgam* expression in EPI fat and *Itgax* expression in ING fat, indicating reduced myeloid cell infiltration in these tissues (Figure 3c). This effect could be mediated by RAPA‐ and RAPA/MET suppression of genes for cytokines such as *Hmox1* (heme oxygenase 1), *Lbp* (lipopolysaccharide binding protein), and *Serpine1* (plasminogen activator inhibitor‐1, *PAI‐1*) in both fat depots (Figure 3b, Table S1), which are key genes for the production of signals that promote inflammatory chemotaxis, systemic inflammation, and insulin resistance (Alessi et al., 2007; De Taeye et al., 2005; Huang et al., 2012).

### 2.5 Insulin resistance, circulating insulin, and IGF‐1

**FIGURE 4:**
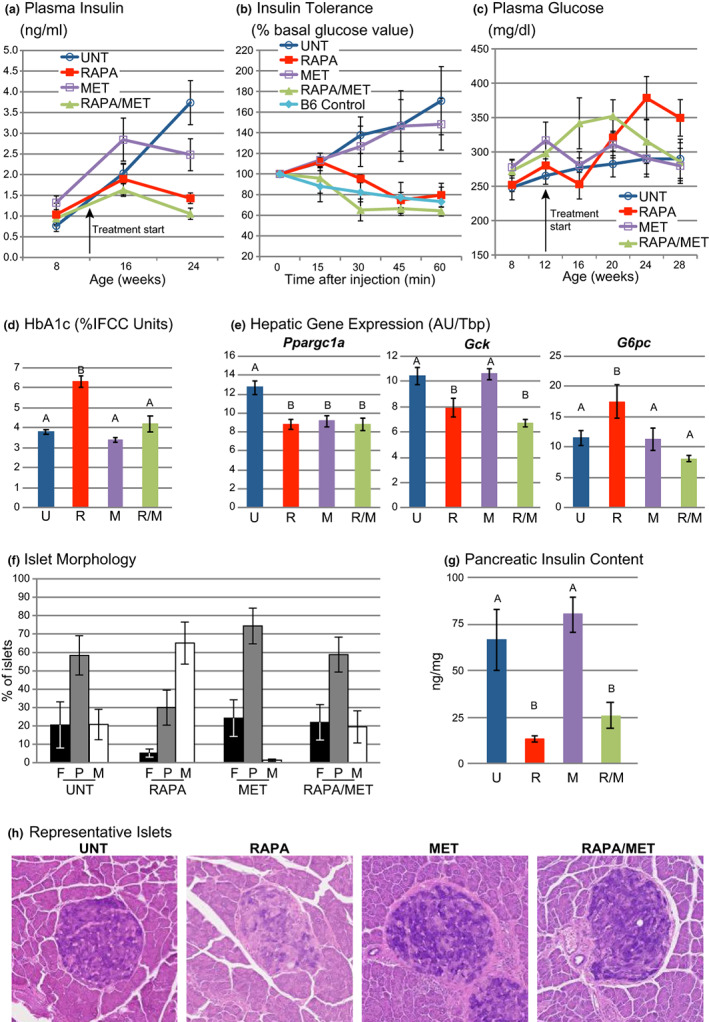
RAPA/MET‐treatment normalized insulin sensitivity and ameliorated the effects of RAPA‐treatment on islet granulation. (a) UNT mice progressed toward hyperinsulinemia. MET‐treatment reduced this hyperinsulinemia (*p* = 0.01 vs. UNT after 12 weeks of treatment, Tukey–Kramer HSD). RAPA‐ and RAPA/MET‐treatment prevented hyperinsulinemia (*p* < 0.0001 for both vs. UNT after 12 weeks of treatment, Tukey–Kramer HSD). (b) UNT mice were insulin resistant, indicated by the insulin tolerance test (ITT, showing the percentage of circulating glucose of the pre‐injection basal level). RAPA‐treatment for 17 weeks improved insulin sensitivity (repeated measures MANOVA, *p* = 0.03 vs. UNT; one RAPA‐treated mouse with glucose values >600 mg/dl at 45 and 60 min after insulin injection was censored as an outlier). MET‐treatment had no effect. RAPA/MET‐treatment normalized insulin sensitivity to a level equivalent to that of untreated C57BL/6J males (repeated measures MANOVA, *p* = 0.005 vs. UNT). (c) UNT mice developed hyperglycemia as expected (Leiter et al., 2013; Leiter & Reifsnyder, 2004). RAPA‐treatment further elevated this hyperglycemia by 12 weeks of treatment (*p* = 0.03, Tukey–Kramer HSD). MET‐treatment had no effect. After treatment was initiated, RAPA/MET‐treatment reduced hyperglycemia compared to RAPA‐treatment alone (repeated measures MANOVA, 16–28 weeks of age, interaction of treatment with time, *p* = 0.03). Glucose levels in RAPA/MET‐treated mice were comparable to levels in UNT mice after 16 weeks of treatment. (d) RAPA/MET‐treatment prevented RAPA‐ driven elevation of HbA1c, measured 18 weeks after treatment was initiated. (e) All three treatments reduced hepatic *Ppargc1a* expression. Reduced hepatic *Gck* and increased *G6pc* expression in RAPA‐treated mice are associated with elevated hyperglycemia. RAPA/MET‐ treatment counteracts this hyperglycemic effect potentially by preventing the rapamycin‐associated elevation of *G6pc*. (f) All islets from 3 separate sections (per pancreas) were scored for granulation status (insulin storage): F = fully‐granulated; P = partially‐degranulated; or M = mostly‐to‐completely‐degranulated. Treatment affected the islet degranulation profile (*p* = 0.0006, MANOVA, Wilk's λ). RAPA‐treatment significantly exacerbated the phenotype (post hoc pairwise comparisons, *p* = 0.02, MANOVA, Wilk's λ). MET‐treatment had no effect versus UNT. RAPA/MET‐treatment prevented the effect of RAPA‐treatment on islet morphology (*p* = 0.03, MANOVA, Wilk's λ). (g) RAPA‐ and RAPA/MET‐treatments reduced pancreatic insulin content. (h) Representative islets illustrate effects of treatment on islet granulation (aldehyde fuchsin staining). The islet from an UNT mouse illustrates partial degranulation. The islet from a RAPA‐treated mouse illustrates the mostly degranulated condition of the majority of islets in RAPA‐treated mice. Islets from the MET‐ and RAPA/MET‐treated mice illustrate the partial degranulation typical of islets from these mice. All histologic pictures are at the same magnification. Mean ± SE. *N* = 9–10 mice per treatment except (b), *N* = 5–6 mice per treatment. Significance annotations and treatment abbreviations as in Figure 1

Plasma insulin increased progressively in untreated males as observed previously (Reifsnyder et al., 2014). While MET‐treatment mitigated this increase after 12 weeks of treatment, both RAPA‐ and RAPA/MET‐treatment completely prevented it (Figure 4a). Insulin tolerance (ITT) at 17 weeks of treatment showed that untreated males were highly insulin resistant, whereas RAPA‐treated males were insulin sensitive (Figure 4b), as observed previously in this and other T2D strains of mice (Reifsnyder et al., 2016). While MET‐treatment had no effect on insulin tolerance or HOMA‐IR, RAPA‐ and RAPA/MET‐ treatment significantly improved HOMA‐IR (Table 2). In fact, RAPA/MET‐treatment completely normalized insulin tolerance to levels comparable with that of the young, insulin sensitive, C57BL/6J control males (Figure 4b). At 16 weeks of treatment circulating insulin‐like growth factor 1 (IGF‐1) was reduced by 23% in RAPA/MET‐treated mice (Table 2).

### 2.6 Plasma glucose (PG) and gluconeogenesis

Untreated NcZ10 males transited to diabetes (glucose >250 mg/dl) from 8 to 16 weeks of age, as previously reported (Leiter et al., 2013; Leiter & Reifsnyder, 2004). RAPA‐treatment significantly increased PG elevation by 12 weeks of treatment (Figure 4c) and circulating HbA1c levels, measured at the end of the 18‐week treatment (Figure 4d). MET‐ treatment had no effect on PG or HbA1c levels compared to untreated mice (Figure 4c,d). While RAPA/MET‐treatment for 8 weeks elevated PG levels, after 16 weeks of treatment, PG, as well as HbA1c level, were decreased to levels observed in untreated mice (Figure 4c,d). Thus, MET eventually prevented the RAPA‐mediated exacerbation of hyperglycemia.

We evaluated the expression of genes that govern gluconeogenesis to identify potential mechanisms by which MET co‐treatment may moderate RAPA‐induced hyperglycemia. At 18 weeks of treatment, all three treatments downregulated hepatic *Ppargc1a* (Figure 4e). Such downregulation is generally associated with diminished gluconeogenesis (Puigserver et al., 2003), yet RAPA‐treatment elevated circulating glucose, compared to controls, measured during a short‐term fast that preceded blood sampling (Figure 4c). RAPA‐treatment reduced hepatic glucokinase (*Gck*) and increased glucose‐6‐phosphatase (*G6pc*) expression (Figure 4e, Table S2), which could reduce phosphorylation of intracellular free glucose and increase release of glucose into circulation (Iynedjian, 2009), contributing to the RAPA‐induced hyperglycemia through an mTORC2‐mediated mechanism (Lamming et al., 2014). While co‐treatment with MET did not affect *Gck* expression, *G6pc* was suppressed, potentially diminishing hepatic release of glucose and contributing to the MET‐mediated prevention of RAPA‐driven hyperglycemia.

### 2.7 Pancreas and pancreatic insulin content (PIC)

By 30 weeks of age, untreated male NcZ10 mice exhibited islet degranulation indicative of diminished insulin storage that is characteristic of an early, hyperinsulinemic phase of T2D (Figure 4f). Overall, treatments produced significant differences in degree of islet degranulation (Figure 4f, MANOVA, *p* = 0.0006, Wilk's λ): Compared to untreated mice, RAPA‐treatment produced a more severe degranulation (MANOVA, *p* = 0.02), and MET‐treatment produced more fully granulated islets (MANOVA, *p* = 0.05). RAPA/MET‐treatment prevented the RAPA‐associated degranulation (*p* = 0.03), maintaining a pattern comparable with that of untreated mice (Figure 4f). RAPA‐ and RAPA/MET‐treatments reduced islet numbers (Table S3) and PIC (Figure 4g); MET‐treatment had no effect on islet number or PIC. No treatment affected islet size distribution (Table S3). Overall, while MET‐treatment prevented the level of islet degranulation produced by RAPA‐treatment, it did not prevent some reduction of islet number and PIC.

### 2.8 Diabetic nephropathy and neuropathy

**FIGURE 5:**
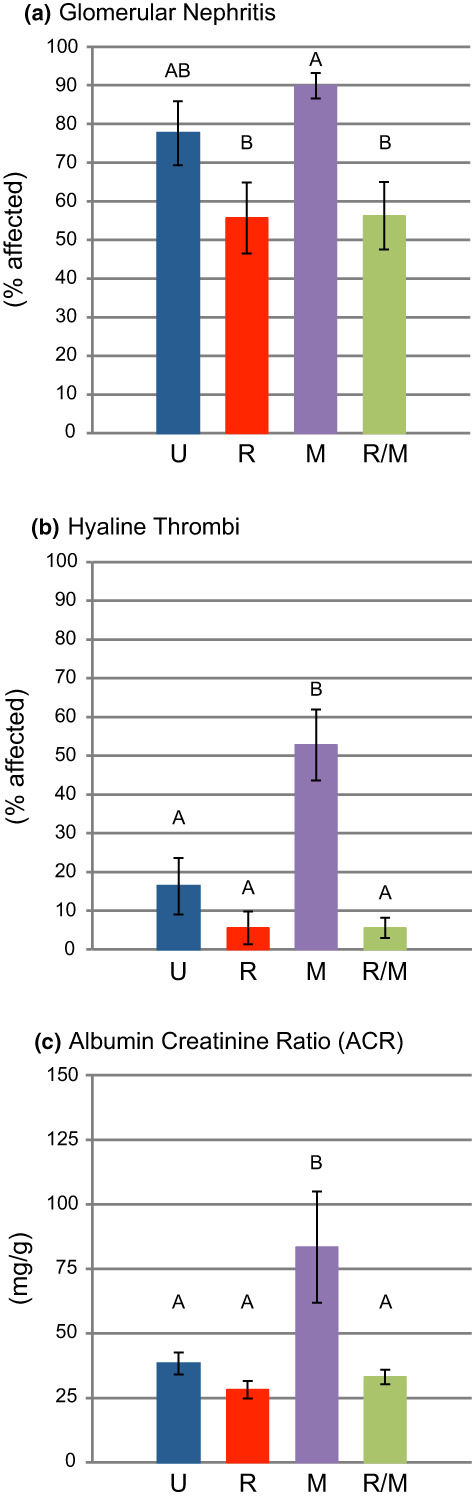
RAPA‐ and RAPA/MET‐treatments prevented the promotion of nephropathy by metformin. (a) Glomerular nephritis was widespread in MET‐treated and UNT mice. Addition of RAPA reduced the incidence of glomerular nephritis compared to that in MET‐treated mice. (b) MET‐treatment increased the incidence of hyaline thrombi; this increase was prevented in RAPA/MET‐treated mice. (c) MET‐treatment doubled the urinary ACR level; RAPA/MET‐treatment prevented this effect of metformin on ACR level. Mean ± SE. *N* = 9–10 mice per treatment. Significance annotations and treatment abbreviations as in Figure 1

Nephritis was observed in ~75% of the glomeruli in untreated males (Figure 5a), with hyaline thrombi observed in ~15% of the glomeruli (Figure 5b). One untreated mouse had an extremely high albumin/creatinine ratio (ACR = 1042, about 25 times normal) and was removed from this analysis as an outlier. Surprisingly, ACR was not elevated in the remaining untreated mice (Figure 5c), although we did observe it in mature NcZ10 males previously (Reifsnyder et al., 2014). RAPA‐treatment had no significant effect on ACR, whereas MET‐treatment increased the proportion of glomeruli with hyaline thrombi and doubled the ACR (Figure 5b,c), consistent with a nephrotoxic potential of metformin (Thomas & Bakris, 2014). Combination RAPA/MET‐treatment completely blocked the nephrotoxic effect of MET (Figure 5a–c).

Intraepidermal small sensory nerve fiber density (IENF) in foot pad skin, identified by immunocytochemistry (Jolivalt et al., 2016), is a standard histologic metric for small‐fiber neuropathy. IENF density did not differ among the 4 groups (Table 2). In mice with T2D, evidence of peripheral neuropathy more typically appears as insulinopenia develops (Jolivalt et al., 2016), which may occur at a later stage of diabetes in NcZ10 mice.

## 3 DISCUSSION

**FIGURE 6:**
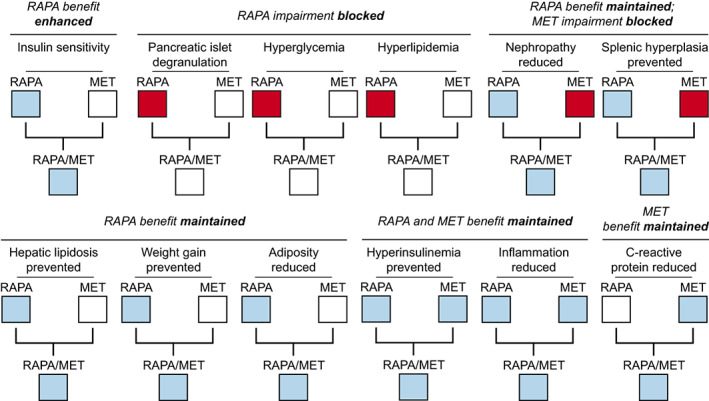
Summary of treatment effects on T2D in the NONcNZO10/LtJ (NcZ10) strain. RAPA/MET‐treatment maintained positive effects of RAPA‐treatment on various aspects of T2D while ameliorating negative side effects of RAPA‐treatment and MET‐treatment alone. White boxes denote no difference between treated versus untreated mice, blue boxes denote beneficial effect in treated versus untreated, red boxes denote negative effect in treated versus untreated mice

Our results demonstrate that it is possible to prevent the hyperglycemic effect of RAPA‐treatment without diminishing its beneficial effects. The addition of MET to the treatment not only blocked the RAPA‐elevated hyperglycemia and hyperlipidemia in NcZ10 mice, but it also protected pancreatic islets from RAPA‐mediated degranulation. This beneficial drug interaction was reciprocal: RAPA blocked a nephrotoxic influence of MET. Importantly, both drugs synergized to generate completely normal systemic insulin sensitivity in this innately insulin resistant NcZ10 model (Figure 6).

### 3.1 Obesity and adipose tissue

Obesity is a primary driver of T2D metabolic dysfunction (Bastard et al., 2006; Johnson, 2021). In two diabesity models of T2D, polygenic NcZ10 males of this study and monogenic C57BLKS/J‐*Lepr* ^*db*^ (BKS‐*db/db*) females (Deepa et al., 2013), rapamycin feeding prevented continued body weight gain by preventing increased adiposity; lean mass was not diminished in either model. The marked RAPA‐mediated downregulation of *Mest* we observed is noteworthy because adipose tissue MEST contributes to fat mass expansion in mice (Anunciado‐Koza et al., 2016; Anunciado‐Koza et al., 2017). The reduced expression of inflammatory and hypoxia‐associated genes in adipose tissue and the improved systemic insulin sensitivity of RAPA‐treated mice are consistent with observations in *Mest*‐deficient mice (Anunciado‐Koza et al., 2017). Our data suggest that the effect of RAPA on obesity and improved systemic insulin sensitivity may be mediated partly through downregulation of MEST‐facilitated fat mass expansion and the related tissue hypoxia and adipose tissue inflammation. In addition, reduced expression of insulin‐sensitive *Gpam* expression in adipose tissue of RAPA‐ and RAPA/MET‐treated mice potentially limits triglyceride synthesis by sustaining lipolysis in the fed state, contributing to the reduction in adipocyte hypertrophy (Gonzalez‐Baro et al., 2007; Yu et al., 2018) while promoting hypertriglyceridemia by interfering with triglyceride clearance (Paolella et al., 2020). The addition of MET to the RAPA‐treatment does not interfere with the inhibitory effect on *Gpam* expression, but it does prevent the consequent promotion of hypertriglyceridemia, potentially through its restoration of hepatic *Ldlr* expression (see “Steatosis and hyperlipidemia” below).

Interestingly, although the expression of both *Mest* and *Gpam* was suppressed in inguinal (ING) fat as well as epidydimal (EPI) fat, EPI fat did not decrease in size (Table 1). This may have resulted from the upregulation of the insulin‐responsive glucose transport gene *Slc2a4* (Glut4) specifically in EPI fat, which presumably led to increased glucose uptake, sustaining tissue mass. Although maintenance of adipose tissue mass would generally maintain insulin resistance, up‐regulation of *Slc2a4* instead would be expected to sensitize the expanding tissue to insulin's effect on glucose uptake. Thus, gene expression analysis suggests that RAPA‐ and RAPA/MET‐treatments generate systemic insulin sensitivity in this inherently insulin‐resistant mouse model through effects on adipose tissue involving at least two mechanisms: by limiting expansion of adipose tissue itself and by sensitizing specific depots to insulin.

*Ucp1, Ppargc1α*, and *Dio2* were unaffected or downregulated by RAPA‐treatment (Figure 1c, Table S1). Thus, it is unlikely that thermogenesis‐associated genes contribute to the RAPA‐ and RAPA/MET‐mediated reductions in white adipose tissue in the mice of our study, which is consistent with reports showing RAPA‐mediated suppression of beigeing/browning of white adipose tissue (Tran et al., 2016).

Diminished adipose tissue expansion primarily reflects effects of RAPA‐treatment. MET‐treatment alone showed no effects on adiposity and minimal changes in the expression of the adipose genes measured. Furthermore, MET, when combined with RAPA, did not alter the effect of RAPA on gene expression in both EPI and ING fat (Table S1).

### 3.2 Steatosis and hyperlipidemia

Rapamycin induces changes in circulating insulin and in hepatic gene expression that can contribute to the prevention of steatosis. Reduced hepatic steatosis in RAPA‐treated type 2 diabetic rats has been observed in association with improved insulin sensitivity (Zhou & Ye, 2018). In our study, protection from pre‐steatotic lipidosis (Figure 2b) paralleled the effect of these treatments on circulating insulin (Figure 4a), which affects hepatic steatosis through regulation of *Fasn* (hepatic fatty acid synthase) expression (Figure 4c) (Liu et al., 2018). *Fasn* codes for a rate‐limiting enzyme for hepatic fatty acid synthesis, and its elevated expression is closely associated with the development of hepatic steatosis in humans and mice (Dorn et al., 2010). Thus, the suppression of hepatic *Fasn* by RAPA‐ and RAPA/MET‐treatment, potentially resulting from reduction of circulating insulin, provides a mechanism by which these treatments can prevent diabetic steatosis.

Hyperlipidemia is a common side effect in patients undergoing treatments that inhibit mTOR (Morrisett et al., 2002). In our study, the reduced levels of hepatic *Ldlr* mRNA in RAPA‐treated mice, and the inhibition of this effect with RAPA/MET‐treatment, corresponded inversely with the effects on circulating triglycerides (Figure 2a,c), presumably due to the important role for LDLR in the clearance of APOB‐ and APOE‐containing lipoproteins.

### 3.3 Inflammation

The development of obesity can generate progressive systemic insulin resistance, which promotes inflammation and aggravation of the metabolic syndrome (Paniagua, 2016; Shimobayashi et al., 2018). In high fat‐fed DBA/1J mice with collagen‐induced rheumatoid arthritis, treatment with the combination of metformin and rapamycin given per os for 10 weeks virtually eliminated joint collagen damage and inflammation (Kim et al., 2020). In the diabetic NcZ10 mice of our study, RAPA/MET‐treatment reduced both the extent and intensity of systemic inflammation (Figure 3a,b). The RAPA‐ and RAPA/MET‐induced reduction in adipose tissue myeloid infiltration and expression of genes for inflammatory stress‐responsive factors and cell senescence (*Itgam, Itgax, Hmox1, Lbp, and Serpine1*) demonstrate that these treatments specifically suppress obesity‐associated inflammation (Figure 3c). A recent study (Li et al., 2021) using human subcutaneous adipocytes suggests that effects of insulin mitogenic signaling on adipocyte cell cycle dynamics are involved. Prolonged elevated insulin exposure can reinitiate cell cycle progression in mature adipocytes; however, hyperinsulinemia promotes premature cell cycle exit leading to a senescent secretory profile, including inflammatory cytokines. Metformin‐treatment inhibited the hyperinsulinemia‐associated induction of adipocyte senescence, and it suppressed the secretion of most proinflammatory cytokines. Rapamycin, through its direct suppression of mTOR signaling, could act through a comparable mechanism. Interestingly, the lack of an effect of RAPA‐ or RAPA/MET‐treatment on EPI fat mass did not prevent beneficial RAPA‐mediated effects on Epi fat inflammatory gene expression, indicating that augmentation of adipose tissue, per se, is insufficient to sustain adipogenic pathogenesis, and that hyperinsulinemic signaling may be a key factor.

### 3.4 Circulating glucose and insulin resistance

NcZ10 mice transit to an obese and hyperglycemic state by 12–13 wk of age (Figures 1a and 4c) in association with the development of insulin resistance in skeletal muscle, liver, and heart (Cho et al., 2007). Because we sampled blood for glucose determination during a short‐term morning fast, the blood glucose level was determined primarily by the rate of hepatic glucose release, the circulating insulin level, and systemic insulin sensitivity. Systemic insulin resistance appears to play no role in RAPA‐generated hyperglycemia in NcZ10 mice as RAPA‐ and RAPA/MET‐treatments actually *reduced* systemic insulin resistance to a level comparable with that of lean, insulin sensitive C57BL6J (Figure 4b). It is noteworthy that rapamycin treatment through the diet has reduced insulin resistance in association with reductions in body weight in a number of mouse models of T2D (Reifsnyder et al., 2016), including BKS‐*db/db* females (Deepa et al., 2013). Instead, the RAPA‐generated hyperglycemia in NcZ10 mice appears to result from effects on hepatic glucose discharge and constrained pancreatic response to circulating glucose levels.

Our gene expression results indicate that hepatic glucose discharge may be affected by effects of RAPA‐ and RAPA/MET‐treatments on hepatic transmembrane glucose transport. RAPA‐treatment shifted the expression ratio of *Gck* and *G6pc* (Figure 4e), key genes for the regulation of intracellular glucose phosphorylation and transmembrane transport (van Schaftingen & Gerin, 2002), predisposing the gene expression profile to favor hepatic glucose export and promote hyperglycemia. This pattern is consistent with the mTORC2‐regulated nutrient effect on *Gck* and *G6pc* (Lamming et al., 2014), and it suggests the possibility that dietary rapamycin holds the potential for mTORC2 activation and promotion of hepatic insulin resistance, at least in mice at risk for T2D such as NcZ10 mice. RAPA/MET‐treatment counteracted the effect that RAPA‐treatment alone had on *G6pc* expression, restoring a more normal ratio with *Gsk*, and it prevented the specific hyperglycemia that was associated with RAPA‐treatment. Thus, the contrary effects of RAPA‐treatment versus RAPA/MET‐treatment on the expression of *G6pc*, a gatekeeper of hepatic glucose discharge, potentially influence the effect these treatments have on diabetic hyperglycemia.

### 3.5 Pancreatic islets and insulin

By 8 weeks of age, NcZ10 males exhibit a histopathologic phenotype characterized by islet hypertrophy. As chronic hyperglycemia develops by 12–13 weeks, islet β‐cell degradation and atrophy are apparent, demonstrating that NcZ10 males present with an atypical degree of islet sensitivity to metabolic stress. By 24 weeks of age islets manifest extensive β‐cell degranulation (Leiter et al., 2013; Leiter & Reifsnyder, 2004), reflecting depletion of insulin storage. Gene expression analyses of pancreases from 22‐week‐old NcZ10 males showed decreased expression of *Slc2a2* (Glut2) mRNA and downregulation of TCA cycle and electron transport genes, suggesting candidate mechanisms of pancreatic impairment in NcZ10 mice (Hirata et al., 2017).

In our study, RAPA‐treatment in NcZ10 males led to further pancreatic islet degranulation, (Figure 4f), and reduced islet number (Table S3), resulting in reduction of total PIC (Figure 4g), compared with untreated NcZ10 males. RAPA/MET‐treatment eventually protected the islet β‐cells from RAPA‐driven hyperglycemic hyperstimulation and tempered the islet degranulation that resulted from RAPA‐treatment, but it did not comparably preserve total islet number and PIC, which may have been affected by the earlier elevation of circulating glucose during the first three months of treatment. Thus, while the prevention of RAPA‐driven hyperglycemia in RAPA/MET‐treated mice may protect pancreatic islets from additional degranulation, the effects of the RAPA‐driven impairment of β‐cell proliferation (Yang et al., 2012) were still evident after 18 weeks of treatment; and, both total islet number (Supplementary Material) and total PIC in RAPA/MET‐treated mice (Figure 4g) were diminished. Rapamycin can also protect the islets from ER stress as demonstrated in the *Ins2* ^−*Akita*^ mutant where rapamycin stimulated autophagy in the pancreatic beta cell thus preventing the accumulation of mis‐folded insulin that leads to programmed cell death (Bachar‐Wikstrom et al., 2013). In addition, combination with metformin is likely to further reduce ER stress by reducing the RAPA‐induced elevation in circulating glucose (Yong, 2021). The balance of positive and negative effects of rapamycin and metformin in a genetically vulnerable model such as the NcZ10 is expressed in PIC.

The RAPA‐driven reduction of PIC serves as a marker, and potential mechanism, for preventing the progressive elevation of circulating insulin in NcZ10 males as T2D advances (Figure 4a). In RAPA‐treated NcZ10 mice, although circulating glucose was elevated at 12 weeks of treatment, above that of untreated diabetic mice (Figure 4c), circulating insulin remained normal, indicating that the pancreatic response to elevated circulating glucose was constrained. Presumably, this constraint resulted from the reduced islet number and insulin content per islet, and it was reflected by the overall reduction in PIC. Similarly, it has been observed that feeding rapamycin to normoglycemic B6D2F1 mice also produced hyperglycemia and *hypo*insulinemia (Yang et al., 2012). While systemic insulin sensitivity was unaffected by treatment in their study, pancreatic insulin release during a glucose tolerance test was diminished. Further studies of rapamycin treatment, using a 3‐week i.p. injection mouse model, indicated that β‐cell response to glucose (GSIS) was unaffected but that islet mass was reduced because β‐cell proliferation was inhibited (Yang et al., 2012). The association of delayed glucose clearance and diminished PIC in RAPA‐treated mice is further demonstrated in a strain survey of response to RAPA‐treatment (Reifsnyder et al., 2020); The six strains in which PIC was reduced by at least 25% all showed delayed glucose clearance, whereas glucose clearance was unaffected in the two strains with no PIC reduction and one strain with a marginal PIC reduction.

The “insulin restriction” that follows rapamycin treatment, while viewed as a rapamycin‐associated pancreatic impairment (Barlow et al., 2013), may also protect from the development of the T2D co‐morbidities that are promoted by T2D‐associated hyperinsulinemia (Crofts et al., 2015; Templeman, Clee, & Johnson, 2017, Templeman, Flibotte, et al., 2017; Reifsnyder et al., 2018; Li et al., 2021; reviewed in Johnson, 2021). However, a hazardous level of hypoinsulinemia may develop in response to RAPA‐treatment in the context of preexisting pancreatic impairment (Barlow et al., 2013; Reifsnyder et al., 2018), and further study of effects of rapamycin and potential co‐treatments on the maintenance and function of pancreatic islets is needed.

The moderating effect of MET‐treatment on RAPA‐induced hyperglycemia in NcZ10 males was not evident until three months of treatment (Figure 4c). Similarly, in a study of HET3 (normoglycemic) mice receiving the same dietary preparation of rapamycin and metformin used in the present study, it was also reported that RAPA/MET‐treatment did not reduce the elevated circulating glucose generated by RAPA‐treatment in female mice until three months of treatment, with no effect in males even after 9 months (Weiss et al., 2018). These studies demonstrate an interaction of sex with the effects of rapamycin and metformin that depends on the model used.

### 3.6 Nephropathy

RAPA/MET‐treatment prevented the advancement of MET‐driven glomerulonephritis, development of hyaline thrombi, and the elevation of ACR that was generated by MET‐treatment alone (Figure 5). In diabetic kidney disease, hyperactivated mTORC1 may be involved in the pathogenesis of podocyte and tubular cell injury (Yasuda‐Yamahara et al., 2021). Suppression of diabetic nephropathy by direct inhibition of mTORC1 in the podocyte of the nephron (Inoki et al., 2011) provides a direct mechanism for the RAPA‐mediated protection. Reduced insulin resistance and reduced hyperinsulinemia also limits kidney disease (Crofts et al., 2015). Thus, RAPA may suppress diabetic nephropathy through both direct inhibition of mTORC1 and suppression of circulating insulin elevation.

### 3.7 Summary

Treatment of male NcZ10 mice with RAPA promoted further hyperglycemia and hypertriglyceridemia above the levels of untreated controls as T2D developed. Co‐treatment with MET blocked this RAPA‐induced hyperglycemia, in association with normalization of systemic insulin resistance and HOMA‐IR. The RAPA‐induced effects on insulin‐sensitive hepatic gene expression (elevation of gluconeogenic *G6pc* and reduction of *Ldlr* expression) indicate that dietary rapamycin in a genetic context of T2D vulnerability, such as that for NcZ10 males, may promote mTORC2‐dependent hepatic insulin resistance as does i.p.‐ injected rapamycin (Lamming et al., 2012, 2014) in normoglycemic mice.

We investigated the potential of metformin, an established antidiabetic treatment, to diminish the hyperglycemic effect of rapamycin. MET‐treatment alone had few moderating effects on the development of diabetes, including no significant effect on systemic insulin sensitivity and circulating glucose. Nonetheless, co‐treatment with MET blocked the RAPA‐induced hyperglycemia and hypertriglyceridemia. The prevention of effects of RAPA on changes in hepatic gene expression that support glucose discharge and elevate circulating triglycerides suggests that MET may counteract rapamycin‐driven hyperglycemia and hypertriglyceridemia by preventing rapamycin‐promoted hepatic insulin resistance. The reduced hyperglycemia in RAPA/MET‐treated mice may also contribute to their improved islet granulation status, compared to mice treated with RAPA alone, by relaxation of adverse glycemic ER stress in β‐cells (Yong, 2021).

In RAPA/MET‐treated mice, glucose was reduced only to the hyperphysiological level present in untreated diabetic NcZ10 mice, despite the generation of normal systemic insulin sensitivity and the dramatic reduction of HOMA‐IR. The low normal levels of circulating insulin in the context of still elevated glucose indicates impaired pancreatic responsiveness to glucose may be involved. The islet degranulation in RAPA‐treated mice, and the diminished islet number and PIC in both RAPA‐ and RAPA/MET‐treated mice, present functional indices for their limited pancreatic response to glucose (Barlow et al., 2013; Yang et al., 2012) that is manifested by diminished circulating insulin. Such an “insulin restriction” state would contribute to the hyperglycemia and hypertriglyceridemia created by RAPA‐treatment, yet maintenance of relatively low PIC (Reifsnyder et al., 2016) and low circulating insulin (Crofts et al., 2015; Johnson, 2021; Klöting & Bluher, 2005; Templeman, Clee, & Johnson, 2017) is often associated with improved metabolic outcomes and reduction of co‐morbidities in chronic disease states. Additional work is needed to resolve the details of the mechanisms involved; adult inactivation of the insulin receptor (IR) in peripheral tissue can shorten lifespan (Merry et al., 2017), whereas targeted inactivation of the IR can lengthen (Klöting & Bluher, 2005) or shorten (Friesen et al., 2016) lifespan, possibly depending on the specificity of the cre‐recombinase used. Furthermore, the induction of insulin resistance and elevation of circulating insulin in mice via disruption of the mTORC2 complex in adipocytes did not limit caloric restriction‐mediated lifespan extension (Yu et al., 2019). Yet, direct reduction in circulating insulin through inactivation of the *Ins1* gene (*Ins1* ^−/−^) and partial inactivation of *Ins2* (*Ins2* ^−/+^) can delay expression of age‐related diseases and increase lifespan (Templeman, Flibotte, et al., 2017). In this study, numerous benefits of RAPA‐treatment in NcZ10 males can be attributed to RAPA‐induced reductions of diabetic hyperinsulinemia, including diminished lipogenesis, hyperlipidemia, hepatic lipidosis, inflammation, glomerulonephritis, and insulin resistance. Thus, rapamycin‐treatment may be viewed as a double‐edged sword: Many of the benefits of rapamycin‐treatment may result from rapamycin‐induced reductions of insulin secretion, even in the context of hyperglycemia and hypertriglyceridemia, yet treatment of individuals with specific insulin signaling impairments (Friesen et al., 2016) or antecedent diminished pancreatic function (Reifsnyder et al., 2016) can be counterproductive. Our results indicate that co‐treatment with metformin can counteract consequences of rapamycin‐driven hepatic insulin resistance and protect pancreatic islets from aspects of rapamycin‐driven pancreatic impairment while sustaining potentially beneficial reduction of circulating levels of insulin.

### 3.8 Conclusion

Multiple preclinical studies indicate that rapamycin, and its analogues, have positive effects on various chronic age‐related impairments, including cardiomyopathy (Das et al., 2014; Reifsnyder et al., 2018); nephropathy (Reifsnyder et al., 2014; Yang et al., 2007); neurodegeneration, including Alzheimer's disease (Van Skike et al., 2021); and osteoporosis (Kneissel et al., 2004). Positive results for such widespread application have encouraged feasibility studies for larger mammals: dogs (Urfer et al., 2017), nonhuman primates (Ross et al., 2015; Ross & Salmon, 2019; Sills et al., 2019), and humans (Kraig et al., 2018). Although broad use of rapamycin as a generalized antiaging treatment has been contraindicated by its immunosuppressive and hyperglycemic effects, recent research indicates that immunosuppressive effects can be attenuated, and potentially reversed, by alternate treatment schedules and lower doses than used for organ transplantation, and by use of various rapalogues (Arriola Apelo et al., 2016; Chen et al., 2009; Mannick et al., 2018; Walters & Cox, 2018). This study demonstrates that the hyperglycemic effects of rapamycin can be managed with antihyperglycemic co‐treatments such as metformin, and that the benefits of rapamycin are enhanced by such co‐treatment. We propose that effects of rapamycin on pathogenic age‐ or disease‐related insulin elevation may be key for its benefits; however, individuals with marginal pancreatic function may be vulnerable to adverse effects due to a hypotrophic influence on islet maintenance. Our results advance consideration of rapamycin‐based treatment strategies that are individually tailored to effects on systemic insulin levels.

## 4 METHODS

### 4.1 Animals, diets, and caging

Forty NONcNZO10/LtJ (NcZ10; JAX® Stock Number 4456) males at 8 weeks of age were transferred from The Jackson Laboratory (Bar Harbor, ME, USA) production facility to the investigator's pathogen‐free mouse room (D1) at The Jackson Laboratory (health status report: http://jaxmice.jax.org/genetichealth/index.html). Mice were fed a chow diet containing 11% fat (5LA0; all diets prepared by Test Diet, Inc., division of Purina Mills, Richmond, IN, USA) until 12 weeks of age. Then groups of 10 mice each were either maintained on the 5LA0 chow diet (untreated), or switched to 5LA0 containing 14 ppm encapsulated rapamycin (RAPA), 0.1% metformin (MET), or both rapamycin (14 ppm) and metformin (0.1%) (RAPA/MET). Rapamycin purchased and encapsulated as per Harrison et al. (2009). Metformin was a gift from Rafael de Cabo (National Institute on Aging Intramural Program). All procedures were approved by the Animal Care and Use Committee of the Jackson Laboratory (Animal Use Summary #99084) and comply with guidelines in accordance with the National Institutes of Health. See online supplementary material for details.

### 4.2 Data collection

Mice were weighed every 2 weeks and bled from the retro‐orbital sinus every 4 weeks. Plasma glucose (PG), plasma insulin (PI), pancreatic insulin content (PIC), c‐reactive protein, plasma insulin‐like growth factor 1 (IGF‐1), urine albumin/creatinine ratio (ACR), insulin tolerance test (ITT), dual x‐ray absorptiometry (DXA, Piximus), food consumption, HbA1c, total and HDL cholesterol, glucose, triglycerides, and nonesterified fatty acids (NEFA) were determined using standard methods (details in supplementary material).

### 4.3 Histology

Pancreas, kidney, liver, spleen, and paw skin were analyzed using standard methods (details in supplementary material). Tissue inflammation was scored as present or absent.

### 4.4 Tissue collection and processing

Liver, epididymal (EPI) fat, and inguinal (ING) fat pads were weighed at termination and quickly frozen in liquid nitrogen prior to storage at −80°C. Gene expression studies used standard methods (details in supplementary material).

### 4.5 Statistics

One‐way ANOVA, repeated measures MANOVA, the Tukey–Kramer procedure for post hoc comparisons, correlation analysis, ordinal logistic and nominal logistic analyses were performed using JMP (SAS Institute, Inc., Cary, NC). Two‐way ANOVA was used for analysis of gene expression (GraphPad Prism V.6, GraphPad Software, Inc., La Jolla, CA) to assess possible interactions of rapamycin and metformin. All analyses were two‐sided with respect to the null hypothesis.

## AUTHOR CONTRIBUTIONS

Peter C. Reifsnyder designed and conducted the experiments, performed data analyses, and wrote the manuscript. Robert A. Koza performed the expression analysis and contributed text to the manuscript. Rosalinda Doty scored the histologic pathology and reviewed the manuscript. Nigel A. Calcutt quantified foot skin IENF and contributed text to the manuscript. Kevin Flurkey performed data analyses and cowrote the manuscript. David E. Harrison suggested the study, contributed to study design and reviewed/edited the manuscript.

## CONFLICT OF INTEREST

The authors have no conflict of interest.

## Supporting information


Appendix S1

Table S1‐S3

## Data Availability

All data generated during and/or analyzed during the current study are available from the corresponding author upon reasonable request.
