# Supplementary material for: Rapamycin/metformin co‐treatment normalizes insulin sensitivity and reduces complications of metabolic syndrome in type 2 diabetic mice
Source: Aging Cell. 2022 Aug 19;21(9):e13666. doi: 10.1111/acel.13666 (PMC9470898; doi:10.1111/acel.13666)
Supplement: Supplementary file 1 — Appendix S1 [file ACEL-21-e13666-s002.docx]

**SUPPLEMENTAL MATERIAL**

**Analysis of relation of islet degranulation to pancreatic insulin content (PIC)**. For analysis of islet degranulation each islet was graded as either fully granulated, 10–50% degranulated (partially), or 50–100% degranulated (mostly). PIC was affected by the degree of islet degranulation even after covariation due to treatment effects (UNT, RAPA-treated, MET-treated, RAPA/MET-treated) was accounted for (MANOVA, *P* = 0.02), indicating that there was a direct relationship of PIC to degree of degranulation. The effect of MET-treatment on the degree of degranulation differed from the other three treatment groups (paired comparisons by MANOVA) (Fig. 4f). To determine if the proportion of islets within a degranulation grade correlated with PIC, analysis of the MET-treated group was separated from the other three groups because the incidence of mostly degranulated islets was so low in MET-treated mice. Among UNT, RAPA-treated and RAPA/MET-treated mice, the proportion of mostly degranulated islets was correlated with PIC, independently of treatment (ANCOVA, *P* = 0.03; the greater the proportion of mostly degranulated islets, the lower the PIC). There was no relationship of the proportion of fully granulated or of partially degranulated islets to PIC among these three treatment groups. For MET-treated mice, there was no overall relation of PIC with relative degree of islet degranulation, probably because there were so few mostly degranulated islets among MET- treated mice (Fig. 4f). We observe comparable relationships of PIC with degranulation if the number, rather than the proportion, of islets in each granulation grade is analyzed; PIC correlated with the number of mostly degranulated islets among the UNT, RAPA-, and RAPA/MET-treated mice, independently of treatment (ANCOVA, *P* = 0.01). Overall, the granulation status of the islets influenced PIC as the proportion of mostly degranulated islets increased.

**Animals, Diets, and Caging**. Forty NONcNZO10/LtJ (NcZ10; JAX® Stock Number 4456) males at eight weeks of age were transferred from The Jackson Laboratory (Bar Harbor, Maine) production facility to the investigator’s pathogen-free mouse room (room D1) at The Jackson Laboratory. Records of pathogens tested are available in health status reports for room D1 at the website: http://jaxmice.jax.org/genetichealth/index.html. The mouse room is maintained on a light/dark cycle of 12 hours, ~25 °C, and 40–50% humidity. All mice were housed in weaning pens (10 mice per 30 cm x 30 cm pen) with pine shaving bedding, given acidified water (pH = 2.5-3.1), and maintained with *ad lib* access to chow diet containing 11% fat (5LA0; all diets prepared by Test Diet, Inc., a division of Purina Mills, Richmond, IN, USA) until 12 weeks of age. At 12 weeks of age groups of 10 mice each were either maintained on the chow diet (5LAO) or switched to 5LAO containing 14 ppm encapsulated rapamycin (RAPA), 0.1% metformin (MET), or both rapamycin (14 ppm) and metformin (0.1%) (RAPA/MET). Rapamycin was purchased and encapsulated as per Harrison, 2009. Metformin was a gift from Rafael de Cabo (National Institute on Aging Intramural Program). A ~40 gram NcZ10 male eats ~3 g of diet per day, thus the treated mice would be consuming roughly 1.1 mg/kg rapamycin and/or 75 mg/kg metformin per day. It is standard protocol for the NcZ10 model to use at least a 10% fat chow diet in order to drive weight gain over the body weight threshold of 36 g to induce diabetes onset (http://jaxmice.jax.org/strain/004456.html).

**Data Collection.** Mice were weighed every 2 weeks and bled every 4 weeks from the retro- orbital sinus. Plasma glucose (PG) values were measured by glucometer (OneTouch, LifeScan, Inc., USA). Plasma insulin (PI) values were measured at 8, 16, and 24 weeks of age by ELISA (Meso Scale Discovery, Gaithersburg, MD, USA). On days that mice were bled, food was removed at 7:00 am (1 hr after lights on) and mice were bled at 10:00–11:00 a.m. Urine samples were collected at 25 weeks of age, and albumin/creatinine ratios (ACRs) were determined using the UniCel DxC 600 Synchron clinical system (Beckman Coulter, Inc., Brea, CA, USA). C-reactive protein (Crystal Chem, Elk Grove Village, IL, USA) and IGF-1 (R&D Systems, Minneapolis, MN, USA) were measured in sera at 28 weeks of age (16 weeks of treatment) by ELISA. The insulin tolerance test (ITT) was performed at 29 weeks of age in a sub-set of each group (N = 5– 6). Food was removed from the mice at 7:00 a.m. At ~10:00–10:30, mice were weighed and bled from the retro-orbital sinus, after desensitization with a drop of tetracaine placed on the eye, and glucose was measured (OneTouch Ultra, Lifescan). Mice were then injected i.p. with 1.0 U/kg insulin (Humulin, Eli Lilly) in PBS. Glucose was measured additionally at 15, 30, 45, and 60 minutes post injection. At 29 weeks, the subset of mice (N = 4) not tested by the ITT was measured for body composition by dual x-ray absorptiometry (DXA, Piximus) while under sedation with tribromoethanol. These mice were then singly housed in metabolic cages (CCMS) and acclimatized for three days prior to measurement of food consumption during the subsequent four days. One RAPA-treated mouse died at 17 weeks of age and all data for this mouse were removed from the study. One untreated and one rapamycin/metformin-treated mouse died at ~25 weeks of age. Mice were euthanized by CO2 at 29–30 weeks of age. HbA1c was determined, using the DxC 600, from whole blood taken at termination. Total and HDL cholesterol, glucose, triglycerides, and non-esterified fatty acids (NEFA) were determined from termination serum using the DxC 600. A handling error precluded the analyses of 2–3 serum samples per group. HOMA-IR was calculated from the insulin and glucose values at 24 weeks of age (fasting insulin concentration in U/ml x fasting glucose concentration in mmol/L divided by 22.5). Conversion of insulin from ng/ml to U/ml: (X ng/ml x 1000 ml/L x 1 mol/5800 g x 1000 pmol)/6 pmol/L/U/ml =Y U/ml. Conversion of glucose from mg/dl to mmol/L: X mg/dl x 0.055 = Y mmol/L.

**Pancreatic insulin content (PIC):** PIC was measured by ELISA (Meso Scale Discovery, Gaithersburg, MD, USA). Half of the pancreas was weighed and frozen in liquid nitrogen at sacrifice. Pancreata were homogenized in acid/ethanol (1.5% HCl, 70% EtOH), and incubated in the acid/ethanol at -20^0^C to extract the insulin. Aliquots of acid/ethanol samples were neutralized in an equal volume of 1M Tris (pH 7.5), then further diluted in deionized water (100-fold; 1:200 final dilution) before running the ELISA.

**Histology.** Pancreas, kidney, liver, spleen and paw skin were collected at termination for histology. Half of the pancreas was fixed in Bouin’s solution; three separate sections were stained with aldehyde fuchsin. Pancreatic histology was assessed by scoring all islets in the three sections for size and degree of granulation: i) fully-granulated, ii) partially-degranulated (10–50%), iii) mostly-degranulated to completely degranulated (50–100%). Kidney, liver, and spleen were fixed in 10% neutral buffered formalin, and separate sections were stained with H&E and PAS. Kidney histology was assessed by scoring 100 glomeruli from each sample for evidence of nephritis and/or hyaline thrombi. Skin was fixed in 4% buffered paraformaldehyde, processed into paraffin blocks, cut, and immunostained with anti-PGP9.5 antibody to visualize IENF and quantified exactly as described elsewhere (Jolivalt, 2016). All scoring was assessed blind to treatment.

**Tissue Collection and Processing for gene expression.** Liver, kidney, epididymal (EPI) fat pad, and inguinal (ING) fat pad tissues were weighed at termination and quickly frozen in liquid nitrogen prior to storage at −80 °C. ING fat pads were taken only from the 16 mice, four per treatment group, that were subjected to DXA and CCMS analyses. RNA was extracted from liver, epididymal fat, and inguinal fat using TRI Reagent (Molecular Research Center Inc. Cincinnati, Ohio), with modifications to remove DNA using the Qiagen RNAeasy columns and DNaseI (Qiagen, Valencia, CA) as previously described (Koza, 2006). RNA was stored at −70 °C in nuclease-free water supplemented with the RNase inhibitor Superasin (Ambion, Austin, TX). Quality and quantity of RNA was determined using UV spectrophotometry (NanoDrop). Quantitative RT-PCR (qRT- PCR) using TaqMan probes and primers and was performed essentially as described (Koza, 2000), except standard curves were generated using pooled RNA from the individual liver or adipose tissue samples used in the study. Gene expression data (arbitrary units; AU) based on the standard curve were normalized to TATA-box binding protein (*Tbp*) for each sample. Probe and primer sequences used to perform qRT-PCR mRNA analyses are available upon request.

Koza, R. A., Hohmann, S. M., Guerra, C., Rossmeisl, M., Kozak, L. P. (2000). Synergistic gene interactions control the induction of the mitochondrial uncoupling protein (Ucp1) gene in white fat tissue. *J. Biol. Chem.* 275(44):34486–34492. doi: 10.1074/jbc.M002136200. PMID: **10931824.**

Koza, R. A., Nikonova, L., Hogan, J., Rim, J. S., Mendoza, T., Faulk, C., Kozak, L. P. (2006). Changes in gene expression foreshadow diet-induced obesity in genetically identical mice. *PLoS Genet.* 2(5), e81. doi: 10.1371/journal.pgen.0020081. Epub 2006 May 26. PMID: **16733553**
