# Supplementary material for: Rapamycin/metformin co‐treatment normalizes insulin sensitivity and reduces complications of metabolic syndrome in type 2 diabetic mice
Source: Aging Cell. 2022 Aug 19;21(9):e13666. doi: 10.1111/acel.13666 (PMC9470898; doi:10.1111/acel.13666)
Supplement: Supplementary file 2 — Table S1‐S3 [file ACEL-21-e13666-s001.docx]

**Supplementary Table 1.** **Adipose gene expression**

|  | **EPI WAT Gene Expression (AU/Tbp; N = 9–10)** | | | | **ING WAT Gene Expression (AU/Tbp; N = 4)** | | | | |
| --- | --- | --- | --- | --- | --- | --- | --- | --- | --- |
| Gene | UNT | RAPA | MET | RAPA/MET | UNT | RAPA | MET | RAPA/MET | |
| ***Innate immunity, inflammation and macrophage infiltration*** | | | | | | | | |  |
| *Lbp* | 15.04 ± 1.70**^a^** | 10.00 ± 0.88**^b^** | 14.44 ± 0.98 **^a^** | 9.79 ± 0.70**^b^** | 6.40 ± 1.18**^a^** | 5.13 ± 0.48**^a^** | 7.85 ± 0.76**^a^** | 5.22 ± 0.43**^a^** | |
| *Itgam* | 12.94 ± 0.84 **^a^** | 9.82 ± 1.75**^b^** | 10.96 ± 0.99 **^b^** | 8.42 ± 0.72**^b^** | 4.06 ± 0.68**^a^** | 3.67 ± 0.33**^a^** | 4.50 ± 0.35**^a^** | 3.66 ± 0.39**^a^** | |
| *Itgax* | 17.51 ± 1.40**^a^** | 8.89 ± 1.90**^b^** | 13.72 ± 1.68**^a^** | 7.16 ± 0.74**^b^** | 3.39 ± 0.50 **^a^** | 1.42 ± 0.13**^b^** | 2.73 ± 0.19**^a^** | 1.33 ± 0.31**^b^** | |
| *Serpine1* | 14.66 ± 1.87**^a^** | 7.68 ± 2.00**^b^** | 9.78 ± 2.11**^ab^** | 6.54 ± 0.97**^b^** | 7.32 ± 1.55**^a^** | 1.25 ± 0.34**^b^** | 5.44 ± 1.04**^a^** | 1.88 ± 0.36**^b^** | |
| *Hmox1* | 16.73 ± 0.95**^a^** | 9.41 ± 1.45**^b^** | 14.44 ± 1.02**^a^** | 9.22 ± 0.67**^b^** | 6.00 ± 1.07**^a^** | 3.72 ± 0.21**^b^** | 7.03 ± 0.52**^a^** | 5.05 ± 0.79**^ab^** | |
| ***Adipose tissue function and fat storage*** | | | | | | | | |  |
| *Mest* | 13.07 ± 0.54**^a^** | 5.84 ± 0.82**^b^** | 12.20 ± 1.36**^a^** | 5.80 ± 0.63**^b^** | 10.93 ± 2.67**^a^** | 3.34 ± 0.57**^b^** | 14.14 ± 2.01**^a^** | 3.46 ± 0.39**^b^** | |
| *Gpam* | 13.66 ± 0.90**^a^** | 10.52 ± 0.75**^b^** | 12.49 ± 0.70**^ab^** | 10.48 ± 0.73**^b^** | 9.05 ± 0.38**^ab^** | 6.79 ± 0.74**^b^** | 10.21 ± 1.01**^a^** | 7.26 ± 0.49**^b^** | |
| *Mrap* | 11.58 ± 0.92**^a^** | 8.45 ± 0.89**^ab^** | 9.54 ± 0.71**^ab^** | 7.22 ± 0.75**^b^** | 10.05 ± 1.26**^a^** | 6.91 ± 0.46**^a^** | 10.44 ± 1.10**^a^** | 6.35 ± 1.25**^a^** | |
| *Lep* | 11.05 ± 0.95**^a^** | 8.98 ± 1.84**^a^** | 8.10 ± 0.53**^a^** | 7.28 ± 1.22**^a^** | 8.32 ± 2.12**^a^** | 3.56 ± 0.61**^b^** | 8.30 ± 0.56**^a^** | 2.36 ± 0.52**^b^** | |
| *Ucp1* | 8.61 ± 2.01**^a^** | 8.74 ± 0.71**^a^** | 8.17 ± 1.79**^a^** | 7.45 ± 1.14**^a^** | 10.79 ± 4.28**^a^** | 10.80 ± 2.43**^a^** | 25.79 ± 13.18**^a^** | 12.01 ± 5.99**^a^** | |
| *Slc2a4* | 4.77 ± 0.60**^a^** | 9.74 ± 0.99**^b^** | 4.49 ± 0.68**^a^** | 9.57 ± 0.80**^b^** | 11.88 ± 1.21**^a^** | 14.07 ± 0.76**^a^** | 13.26 ± 1.84**^a^** | 11.74 ± 0.99**^a^** | |
| ***Mitochondrial biogenesis, angiogenesis and thyroid hormone regulation*** | | | | | | | | |  |
| *Ppargc1a* | 5.52 ± 0.97**^a^** | 6.30 ± 0.96**^a^** | 5.83 ± 0.87**^a^** | 6.26 ± 0.96**^a^** | 13.93 ± 2.40**^a^** | 19.95 ± 0.62**^a^** | 14.95 ± 0.58**^a^** | 18.02 ± 1.56**^a^** | |
| *Vegfa* | 7.31 ± 0.50**^a^** | 10.04 ± 0.81**^b^** | 7.17 ± 0.65**^a^** | 10.80 ± 0.42**^b^** | 11.96 ± 1.29**^a^** | 14.48 ± 1.20**^a^** | 15.51 ± 1.49**^a^** | 14.66 ± 2.02**^a^** | |
| *Dio2* | 16.28 ± 1.75**^a^** | 6.93 ± 0.95**^b^** | 15.05 ± 1.13**^a^** | 6.67 ± 0.94**^b^** | 14.56 ± 3.09**^a^** | 2.12 ± 0.22**^b^** | 9.78 ± 1.64**^a^** | 2.07 ± 0.14**^b^** | |

Mean ± SE. 2-way ANOVA analyses of gene expression in epididymal (EPI) and inguinal (ING) white adipose tissue (WAT) of NcZ10 mice. Untreated = UNT, RAPA-treated = RAPA, MET-treated = MET, RAPA/MET-treated = RAPA/MET. Results for treatments not annotated by the same superscript letter are significantly different at *P* < 0.05 (Tukey-Kramer HSD). There were no significant interactions of RAPA with MET.

**Supplementary Table 2**. **Hepatic gene expression**

|  | **Hepatic Gene Expression (AU/Tbp; N = 9–10)** | | | | | **Interaction effect** |
| --- | --- | --- | --- | --- | --- | --- |
| Gene | UNT | RAPA | MET | RAPA/MET | RAPA x MET | |
| *Fasn* | 11.28 ± 1.01**^a^** | 8.88 ± 0.93**^b^** | 8.58 ± 0.60**^b^** | 8.15 ± 0.66**^b^** | n.s. | |
| *G6pc* | 11.52 ± 1.31**^a^** | 17.54 ± 2.80**^b^** | 11.31 ± 1.74**^a^** | 8.08 ± 0.51**^a^** | n.s. | |
| *Gck* | 10.45 ± 0.68**^a^** | 7.93 ± 0.69**^b^** | 10.58 ± 0.40**^a^** | 6.72 ± 0.31**^b^** | n.s. | |
| *Ldlr* | 10.28 ± 0.48**^a^** | 7.52 ± 0.36**^b^** | 10.28 ± 0.22**^a^** | 9.34 ± 0.52**^a^** | *P* = 0.03 | |
| *Ppara* | 9.25 ± 0.69**^a^** | 7.66 ± 0.48**^a^** | 9.83 ± 0.46**^ab^** | 10.89 ± 0.55**^b^** | *P* = 0.02 | |
| *Ppard* | 7.44 ± 0.96**^ab^** | 15.18 ± 3.38**^a^** | 10.60 ± 3.00**^ab^** | 5.08 ± 0.80**^b^** | *P* = 0.009 | |
| *Ppargc1a* | 12.69 ± 0.75**^a^** | 8.79 ± 0.53**^b^** | 9.19 ± 0.59**^b^** | 8.83 ± 0.68**^b^** | *P* = 0.009 | |
| *Slc2a2* | 10.67 ± 0.91**^a^** | 10.01 ± 0.49**^ab^** | 8.05 ± 0.41**^b^** | 8.86 ± 0.67**^ab^** | n.s. | |

Mean ± SE. 2-way ANOVA analyses of hepatic gene expression in NcZ10 mice. Untreated = UNT, RAPA-treated = RAPA, MET-treated = MET, RAPA/MET-treated = RAPA/MET. Results for treatments not annotated by the same superscript letter are significantly different at *P* < 0.05 (Tukey-Kramer HSD).

**Supplementary Table 3, Islet numbers**

| Treatment | n | # Small (%) | # Medium (%) | # Large (%) | # Total |
| --- | --- | --- | --- | --- | --- |
| UNT | 9 | 31.2 ± 4.7 (50) | 21.1 ± 1.9 (34) | 9.4 ± 1.6 (15) | 61.8 ± 5.4**^a^** |
| RAPA | 9 | 18.2 ± 2.8 (56) | 11.2 ± 1.9 (35) | 2.9 ± 0.9 (9) | 32.3 ± 3.9**^c^** |
| MET | 10 | 37.0 ± 3.9 (64) | 15.7 ± 2.6 (27) | 5.3 ± 1.4 (9) | 58.0 ± 5.9**^ab^** |
| RAPA/MET | 9 | 27.0 ± 2.3 (62) | 11.9 ± 1.4 (27) | 4.6 ± 1.2 (11) | 43.4 ± 2.4**^bc^** |

Mean ± SE. While there are significant differences in the total numbers of islets between the groups (results for treatments not annotated by the same superscript letter are significantly different at *P* < 0.05 by Tukey-Kramer HSD), the size spectrum of islets is unchanged.
